# Enhancing Psychological Sexual Health of People With Spinal Cord Injury and Their Partners in an Italian Unipolar Spinal Unit: A Pilot Data Study

**DOI:** 10.3389/fpsyg.2019.00754

**Published:** 2019-04-05

**Authors:** Stefano Federici, Francesco Artegiani, Martina Pigliautile, Paolo Antonelli, Daniele Diotallevi, Innocenza Ritacco, Renée Maschke

**Affiliations:** ^1^Department of Philosophy, Social & Human Sciences and Education, University of Perugia, Perugia, Italy; ^2^MenteCorpo – Clinical Center of Psychology and Sexology, Perugia, Italy; ^3^Gerontology and Geriatrics Section, Department of Medicine, University of Perugia, Perugia, Italy; ^4^Department of Health Sciences, University of Florence, Florence, Italy; ^5^Independent Researcher, Perugia, Italy; ^6^Unipolar Spinal Unit, Hospital Santa Maria della Misericordia, Perugia, Italy

**Keywords:** spinal cord injury, sexuality, sex stereotypes, biopsychosocial model, sexuality and disability, people with paraplegia

## Abstract

Like the slogan of the American Consortium for Spinal Cord Medicine says, “No injury, no matter how serious, can take away your ability to have a relationship, experience love, and experience the attraction between two people.” However, people with spinal cord injury (SCI) have to fight with their own and societal attitudes and stereotypes that limit sexuality to the physiological functions of genitalia, phallocentric primacy of sexual pleasure, and sexual attractiveness of perfect bodies. The scope of the present study is to evaluate the impacts of a psychoeducational intervention in a personal growth group on the sexual life of seven people with SCI and four of their partners particularly their sexual interest and satisfaction, depression, and anxiety. Due to the small samples size, nonparametric statistical tests were used to compare pre- and post-outcome measures for all participants: patients and the partners group. Possible effects of gender were also considered. The effectiveness of the psychoeducational intervention was clearly apparent, showing a high effect size in improving sexual interest and satisfaction, and the opportunity and ability to enjoy sexuality. A reduction of anxiety was also observed for all participants, although it may not have been related to the psychoeducational intervention. Moreover, the intervention significantly improved the opportunity and ability to enjoy sexuality for the partner and patient group. No effect was found on depression. This was the first initiative in Italy aiming to address the sexual life and not only to cure the sexual dysfunction of people with SCI. The positive, clear evidence of the validity of the Love & Life project’s intervention bodes well for new psychoeducational initiatives that in Italy meet the sexual needs of people with SCI and their partners, providing adequate education and psychological support, involving partners, and creating a space to talk among peers.

## Introduction

Can a spinal cord injury (SCI) take away the ability to have a relationship, experience love, and experience the attraction between two people? “No,” says the American Consortium for Spinal Cord Medicine (2011), who made this issue a slogan to promote sexuality and reproductive health in adults with SCI. However, people with SCI have to face many challenges, although not insurmountable (Bodner, 2013), to regain confidence in themselves, in their ability to experience and give pleasure with their body, and in their ability to experience intimacy and affection (Taleporos and McCabe, 2001). After SCI, the change in or loss of genital sensation has a significant impact on sexual experience for both men and women (Chhabra, 2015). In males, the changes are typically related to erection, ejaculation, and orgasm. In women, changes in sexual function include decreased ability to lubricate and reach orgasm (Hammond and Burns, 2009).

Like many other people with disabilities, people with SCI have to fight with their own and societal attitudes and stereotypes denying that individuals with disabilities are sexual beings (Tepper, 2005; Hammond and Burns, 2009). These attitudes and stereotypes are consequences of two prevalent and interrelated myths that very often inform human thinking and behavior: the myth of bodily perfection (Stone, 1995) and the myth of asexuality (Tremain, 1996; Tepper, 1999; Milligan and Neufeldt, 2001; Thompson et al., 2001). These two myths emerge from a model of disability that in literature is often individuated as the medical model of disability (Altman, 2001; Bickenbach, 2012). A model of disability is a categorical representation from which social relations are developed, built, and understood (Meloni et al., 2015). It is a common structure for making sense of the complex phenomena of disability, by helping people to identify and explain social reaction to human, biological, and social diversity. It offers a social frame (Goffman, 1963) in which expected behaviors and social identities are represented, helping people to make decisions and judgments (Federici and Meloni, 2009).

The medical model sees disability as an individual inability to conform to a standard of normality, namely when an individual change occurs within the person (Nagi, 1964). Disability is thus recognizable as that which makes one different from the majority of people (Friedman and Owen, 2017). For instance, in the International Classification of Impairments, Disabilities, and Handicaps, the World Health Organization (World Health Organization [WHO], 1980) defined disability as “any restriction or lack (resulting from an impairment) of ability to perform an activity in the manner or within the range considered normal for a human being” (p. 28). According to this (medical) model of disability, people with SCI have a disability in sexual relations because of the restriction or lack (resulting from the injury) of ability to perform sexual activity in the manner considered normal or ideal (Thomson, 1997). The interrelation between bodily perfection and sexual activity are strictly and clearly related here. “Normal” people must see the individual with SCI as asexual, since the capacity to perform a normal sexual activity is evidently compromised by the injury. For a “normal” population, to conceive of sexual activity by a person with a disability would be to admit figuring out an abnormal (monstrous) sexuality (O’Toole and Bregante, 1992; Tremain, 1996).

The myths of bodily perfection and of the asexuality of persons with a disability are not mere social constructions that inform attitudes and stereotypes. Every cultural context and historical period includes an ideal of bodily perfection and norms about sexual behavior (Wendell, 1996). Therefore, we might consider these myths to be universal human beliefs (Brown, 1991) that emerged from psychological mechanisms evolved to solve long-enduring adaptive problems characteristic of the ancestral human environment (Tooby and Cosmides, 1992). Mating with someone who is unhealthy would have posed a number of adaptive risks for our ancestors, among which were transferring communicable diseases or viruses, impairing survival and reproduction, infecting the children of the union, and imperiling the children’s chances of surviving and reproducing (Buss, 2012, 2016). Hence, an evolved psychological mechanism to avoid contact and sexual intercourse with individuals with visible deformity guaranteed human survival (Tybur et al., 2013; Rozin and Todd, 2016). Park et al. (2003) found that individual differences in concerns about disease predict immediate cognitive responses that link physical disability to disease (medical model), and also predict behavioral avoidance (disgust) of people with physical disabilities. Meloni et al. (2012) also found a link between an evolved mechanism of avoidance of disease and contemporary prejudices affecting people with physical disabilities.

Another clue that the medical model of disability might operate as an evolved psychological mechanism below the two cultural myths of the bodily perfection and asexuality, as a cognitive constraint (Boyer, 1994), is the presence of disability representations consistent with the medical model early in childhood (Smith and Williams, 2004; Huckstadt and Shutts, 2014; Federici et al., 2017), independent of parents’ disability explanations and representations (Meloni et al., 2015).

As nondisabled people, most people with SCI grew up believing that disability is deviance and bodily perfection is the norm (Barnartt, 2010). As Susan Wendell testified in her famous book *The Rejected Body* (Wendell, 1996), recognizing themselves as disabled requires a change in self-identity and adopting a radically new way of thinking about the self. The SCI is nearly always a devastating event with many life-altering implications, which require a variety of adjustment issues throughout post-injury lives (Taleporos and McCabe, 2003; Burns et al., 2008). Sexual adjustment to SCI is one such issue that is intimately related to body image (Taleporos and McCabe, 2001), general psychological health, an underlying sense of self-esteem (Romeo et al., 1993; McCabe et al., 2003), and attractiveness of the body (Kettl et al., 1991; Milligan and Neufeldt, 2001).

In a male-centered patriarchal culture (Code, 2002)—characterized by hierarchal relations between men and women (polarization), with an unequal distribution of power (androcentrism) and biological essentialism (i.e., the difference between sexes and roles are according to nature) (Bem, 1993)—the sex that most people learn about is entirely phallocentric (penis-centered). As Sigmund Freud theorized, the presence or absence of a penis characterizes the sexual identity of people, in such a way that for most men a penis is “a proud possession” (Freud, 1924/1959), and for women the absence of a penis makes them “victim to envy for the penis” (Freud, 1925/1959). This penis orientation has to do with awareness that having and using erections has something to do with masculinity (Zilbergeld, 2013). Therefore, “males are in constant danger of losing their manhood and their identities” (Zilbergeld, 2013) when the erectile functions are compromised. As Mitchell S. Tepper, a sexuality educator and counselor living with SCI, testified,

“[T]he man with erectile dysfunction, inhibited ejaculation, loss of sensation, or physical limitations might conclude that his sex life is over. Attempts at sex may lead to frustration or anger and to relationship conflict. Even if a man is able to attain an erection and ejaculate, he may be disappointed if his erection isn’t as firm or if his ejaculate doesn’t shoot as far as compared to his pre-disability performance. […] When sexual functioning is lost or impaired, men will need help in redefining their concept of good sex and of manhood to be capable of finding enjoyment in their sexuality” [(Tepper, 1999), p. 45].

While male sexuality is more often discussed, with extensive research on erectile functions, female sexuality has been largely ignored (Kettl et al., 1991; Ferreiro-Velasco et al., 2004; Lombardi et al., 2010). This is not surprising within Judeo-Christian androcentrism that restricts the sexual role of the woman to the reproductive function within the family and to the ability to stimulate and to satisfy man’s own sexual appetite (Bem, 1993), denying women the experience of sexual pleasure (Ranke-Heinemann, 1990). Because SCI does not compromise the receptive function of female sexual organs, nor a woman’s reproductive capacity (Consortium for Spinal Cord Medicine, 2011), the biggest issues for women after SCI is usually focused just on the perceived attractiveness of their bodies (Kettl et al., 1991), that is, as a function of male sexuality. In an androcentric, penis-centered sense of sexuality—that Shakespeare et al. (1996) defined as the “fucking ideology” of sexuality: heterosexual penetrative intercourse with a male on top of a female—the loss of genital sensation does not compromise the sexual role of a woman according to Judeo-Christian androcentrism. Far from it, the loss of genital sensation ensures the woman’s virginal and chaste role (Ranke-Heinemann, 1990) (see also the traditional practice of female genital mutilation in Islamic cultures that involves more than 200 million girls and women in 30 countries worldwide^1^. Other evidence that women have a different focus on sex than men, oriented to giving rather than receiving pleasure, includes the studies by M. Alexander and Rosen (2008) and Komisaruk and Whipple (2011) on the orgasm of women with SCI: the major sexual concern of women is the diminishing opportunity and ability to give her partner sexual fulfillment, because the disability disfigured their bodily perfection. Moreover, according to Kettl et al. (1991), “The biggest and most remarkable change [for women] after SCI in our study was the worsening in body image. This was far greater than any change in ratings of sexual practice or enjoyment” (p. 294).

More recent literature (e.g., Sramkova et al., 2017; Thrussell et al., 2018) would seem to shed light on issues other than women’s body image and provide an alternative view to earlier literature, albeit not entirely in contrast. For instance, Sramkova et al. (2017) found that, in women with SCI, compared with the period before the injury, there was a significant decrease in sexual desire, vaginal lubrication, and orgasmic capacity after SCI. However, in the questionnaire used to investigate sexual function after SCI, there was no item to investigate body image, self-esteem, or physical attractiveness. Thrussell et al. (2018), in accordance with previous literature, reaffirm that for women with SCI in the United Kingdom “satisfaction with body image was reduced. To look “sexy” was difficult […]. Lacking confidence and feeling sexually unattractive during rehabilitation was common; support and opportunities to improve self-confidence, self-esteem, body image, and social skills were identified as essential” (pp. 1088–1091).

The purpose of the present study is to provide pilot data for the project Love & Life. The project was carried out in the Unipolar Spinal Unit of the “S. Maria della Misericordia” hospital in Perugia (USU-PG). This unit is one of the twenty-two spinal units belonging to the Italian National Health System, of which nine are Unipolar. Love & Life aimed to enhance the psychological sexual health of USU-PG’s in- and outpatients and their partners. To pursue this goal, the project embraces the World Health Organization’s biopsychosocial model of sexual health (World Health Organization [WHO], 2001, 2006, 2010) and human functioning. This positive, holistic, and comprehensive view states that sexual health is “a state of physical, emotional, mental and social well-being in relation to sexuality; it is not merely the absence of disease, dysfunction or infirmity” (World Health Organization [WHO], 2010, p. 3). This implies overcoming the reductive view of the medical model of human functioning and its social-cultural products (myths, attitudes, stereotypes, and prejudices) that limit sexuality to the physiological functions of genitalia, phallocentric primacy of sexual pleasure, and sexual attractiveness of only bodily perfection. To facilitate a psychological (emotional and behavioral) adjustment that improves self- and sexual esteem as well as satisfaction, the project Love & Life promotes a psychoeducational personal growth group (Nugent, 2013), in which people with SCI and their partners can experience, express, and rework thoughts, fantasies, desires, beliefs, attitudes, values, behaviors, roles, and relationships about sexuality (2010).

## Materials and Methods

### Study Design

The present study is an interventional study to evaluate the impacts of a psychoeducational intervention in a personal growth group on the sexual life of people with SCI and their partners regarding their sexual interest and satisfaction, depression, and anxiety.

### Setting

The inpatients were recruited from the USU-PG between November and December 2017 by the psychologist (IR) employed in the unit. All the inpatients admitted to the USU-PG received a brochure with the aim of the Love & Life project, the criteria for participation, the topics of the activities in the personal growth group on sexual life, the schedule of meetings, and the names and phone numbers of the leaders. The same contents were also posted in the corridors of the unit and of other departments of the hospital in a poster format. IR also recruited the outpatients from the patient registry of the USU-PG by phone during the same period. The personal growth group on sexual life met from December 2017 to May 2018 in the rehabilitation room of the USU-PG, accessible to wheelchairs.

### Participants

The eligibility criteria for attending the Love & Life personal growth group on sexual life were the following:

• Age equal to or greater than 18 years;
• Provide voluntary written informed consent;
• In- and outpatients of USU-PG with a traumatic SCI (para- or tetraplegic), with or without a partner;
• Current partner (wife, husband, sexual partner) of in- and outpatients of USU-PG who attended the Love & Life personal growth group.

We use “participants” to refer to all those who attended the growth group on sexual life and “patients” to refer to both inpatients and outpatients. Fourteen participants attended the growth group on sexual life from December 2017 to May 2018. Only eleven participants were included in the present study, as explained below in the sub-section “Sample” and in the section “Discussion.”

### Measurements and Procedures

A socio-demographic questionnaire and three outcome measures were self-administered (see below) by participants and their partners who had provided voluntary written informed consent during the recruitment process. The outcome measures were administered again at the end of the last group meeting.

#### Socio-Demographic Questionnaire

This was developed *ad hoc* to collect data on participants’ age, gender, sexual orientation, type of SCI (para- or tetraplegia), civil status, children, education, employment, citizenship, political orientation, and religious beliefs. The sexual orientation was rated on the Kinsey scale (Kinsey et al., 1948), also called the Heterosexual-Homosexual Rating Scale^2^. It ranges from 0 to 6, with “0” indicating exclusively heterosexual/opposite sex behavior or attraction and “6” indicating exclusively homosexual/same-sex behavior or attraction. Ratings 1–5 are for those who report varying levels of attraction or sexual activity with either sex. The additional category “X,” which the original Kinsey Report included (Kinsey et al., 1948), designating no socio-sexual contacts or reactions, was not introduced in the questionnaire. The socio-demographic questionnaire was administered to all participants once, before the start of the first group meeting.

#### Sexual Interest and Satisfaction (SIS) Scale

This is a six-item scale, designed to measure sexual adjustment after SCI (Siösteen et al., 1990). It is designed to assess interest in and satisfaction with sexuality before and after injury (Table 1). Partners of the participants with SCI were instructed to answer the questions by making reference to before and after injury of their partners. Participants are asked to give answers on a scale of 0 (non-existent/very dissatisfying) to 3 (increased/very satisfying). It is one of the few sexuality scales that has been used within the SCI population (Abramson et al., 2008). Only one study (Siösteen et al., 1990) reported validity and reliability properties of the scale on a sample of 73 SCI subjects (60 male; mixed injury types; SCI duration >1 year). The SIS scale showed a high correlation with age at injury and moderate to high correlation with quality of life, and a high internal consistency (Cronbach’s α = 0.96).

**Table 1:** Sexual Interest and Satisfaction (SIS) Scale: Domains and underlying questions.

Domains	Questions	Scale point	Score
Sexual desire	1. How is your sexual desire now compared to before injury?	Increased, unchanged, decreased, non-existent	3–0
Importance of sexuality	2. How important is sexuality to you now compared to before injury?	Increased, unchanged, decreased, non-existent	3–0
General satisfaction with sex life after injury	3. How is your relationship, most of the time, with your sexual partner after injury?	Very dissatisfying, partly dissatisfying, partly satisfying, very satisfying	0–3
General satisfaction with sex life before injury	4. How was your relationship, most of the time, with your sexual partner before injury?	Very dissatisfying, partly dissatisfying, partly satisfying, very satisfying	0–3
Self-perceived personal satisfaction	5. How are your opportunity and your ability to enjoy sexuality yourself?	Very dissatisfying, partly dissatisfying, partly satisfying, very satisfying	0–3
Self-rated ability to give partner satisfaction	6. How are your opportunity and your ability to give your partner sexual fulfillment?	Very dissatisfying, partly dissatisfying, partly satisfying, very satisfying	0–3
SIS Scale	Composite of questions	Summary of scale points	0–18
General satisfaction after injury compared with before injury (self-perceived personal satisfaction)		Difference between post- (item 3) and pre-injury (item 4) values	Negative values (<0): more satisfying before injury; Positive values (>0): more satisfying after injury

#### Beck Depression Inventory – II (BDI-II)

In its current version, the BDI-II is a 21-question multiple-choice self-report inventory, composed of items relating to symptoms of depression such as hopelessness and irritability, cognitions such as guilt or feelings of being punished, and physical symptoms such as fatigue, weight loss, and lack of interest in sex (Beck, 2012). Scores for statements ranged from 0 (e.g., “I do not feel sad”) to 3 (e.g., “I am so sad or unhappy that I can’t stand it”). Higher total scores indicate more severe depressive symptoms. The reliability and validity of the BDI-II in Italian have been demonstrated (Beck, 2012).

#### Beck Anxiety Inventory (BAI)

This was designed to differentiate anxiety from depression (Beck and Steer, 1993). Respondents indicate how much they have been bothered by each of 21 symptoms during the past week. Symptoms include the inability to relax and trembling hands. Respondents rated each symptom on a scale ranging from “not at all” (0) to “severely” (3). The reliability and validity of the Italian BAI have been demonstrated (Beck and Steer, 2007).

### Structure, Content, and Techniques of the Psychoeducational Intervention

The personal growth group met on a fortnightly basis for a total of twelve meetings, each lasting two hours, and was conducted by psychologists and psychotherapists proficient in sexuality and disability. The group meetings were structured into two parts: informative and practical. In the informative part, six topics were addressed, each for two meetings: (i) Me and my new body, (ii) Affective-relational communication, (iii) Between identity and sexual orientation, (iv) Discovering pleasure, (v) Live sexual life, and (vi) Aids to pleasure. The content was also conveyed by the projection of sexually explicit videos and images based on the premise that observational learning serves both an informative and a motivational function (Bandura, 1986; Alexander and Sipski, 1993; Tepper, 1997a,c). The interactive practical part, dedicated to personal growth and body awareness, made use of Cognitive-Behavioral therapy, Gestalt therapy, and Emotion-Focused therapy techniques. Through the Cognitive-Behavioral therapy techniques (Hollon and Beck, 2013) (e.g., problem management, role-playing, imagery, modeling) the participants were guided to overcome misconception and negative myths about sexuality and disability, masturbation, orgasm, sexual fantasy, sexual identity, and the beauty and attractiveness of the body through a process of drawing up thoughts and emotions associated with their own sexuality. This process included the identification of possible dysfunctional patterns of self that negatively influenced the relationship with their partners. This implied overcoming the reductive view, stemming from both the medical model of disability, which limits sexuality to physiological functions of genitalia and genital sensation as the only possibility for the sexual experience, and heterosexism. Through Gestalt therapy (Perls et al., 1951) and Emotion-Focused therapy techniques (Elliott and Greenberg, 2017), the creation of a new thinking on sexuality was strengthened by focusing awareness on bodily feelings, emphasizing both the relationship and the process of reflection on aroused emotions to create new meaning. For instance, we used an empty chair and participants’ imagination to invent and guide dialogues to help participants integrate conflicting aspects of their experience, paying attention to the body and verbal language, focusing on feelings and the here and now of the relationship with the therapist or other group members. In the Supplementary Material, two topic guides are provided as an example of two group meetings on sexual life.

### Statistical Analysis

All data were processed using the software IBM SPSS Statistics for Windows, Version 25, Armonk, NY. Due to the small samples size, nonparametric statistical tests were used. Specifically, the Wilcoxon test for paired samples was used to compare pre- and post-intervention scores on SIS, BDI-II, and BAI for the complete sample (participants) and for the two sub-groups of patients and partners.

To find the effect size of the intervention, *r* was used, which was calculated by dividing the *z* value by the square root of *N* (number of cases used in the analysis). The interpretation of *r* values for effect size is relatively similar to Cohen’s *d* (1992). It was considered negligible if it was less than 0.10, small from 0.10 to 0.30, medium between 0.30 and 0.50, and high if it was greater than 0.50. Although we used nonparametric statistics for the analyses, means and standard deviations of the variables (instead of the median) were reported in the tables whenever possible. An independent sample Kolmogorov-Smirnov test was also used to compare the patients and partners groups and to consider possible effects of gender.

## Results

### Sample

Three male participants out of fourteen who signed the informed consent and took part in the group meetings did not complete the entire socio-demographic questionnaire and/or the outcome measures. Therefore, they were excluded from data analyses. Of the remain eleven participants (female: *n* = 6, 54.5%; male: *n* = 5, 45.5%), four males were with complete paraplegia, one female with complete tetraplegia, and one female and male each with incomplete paraplegia. All of them were outpatients during group activity. For all, the cause of SCI was traumatic (years from injury: *M* = 38.1; min = 26; max = 50; *SD* = 9.44). All four partners of the participants with SCI were females. The 11 group participants included four couples (eight individuals). One female participant with SCI reported not having a romantic or sexual partner. Table 2 shows characteristics and statistics of participants collected with the socio-demographic questionnaire.

**Table 2:** Sample profile: Case data are reported in the columns.

	Couple 1		Couple 2		Couple 3		Couple 4		Single 1	Single 2	Single 3
**Sex**	Male	Female	Male	Female	Female	Female	Male	Female	Male	Male	Female
female (*n* = 6, 54.5%); male (*n* = 5, 45.5%)											

**Age**	58	49	39	30	33	32	54	48	48	53	57
female (*M* = 41.5; min = 30; max = 57; *SD* = 11.26) male (*M* = 50.4; min = 39; max = 58; *SD* = 7.3)											

Type of injury	Complete paraplegia	Not paraplegic	Complete paraplegia	Not paraplegic	Incomplete tetraplegia	Not paraplegic	Complete paraplegia	Not paraplegic	Incomplete tetraplegia	Complete paraplegia	Complete tetraplegia
Complete paraplegia (male = 4); Complete tetraplegia (female = 1); Incomplete tetraplegia (female = 1; male = 1)											

Causes of the injury	Traumatic		Traumatic		Traumatic		Traumatic		Traumatic	Traumatic	Traumatic

Years from injury	15		13		3		4		16	4	20
(*M* = 38.1; min = 26; max = 50; *SD* = 9.44)											

Sexual orientation	Hetero	Hetero	Hetero	Hetero	Homo	Homo	Hetero	Hetero	Hetero	Hetero	Predomi- nantly hetero

3 heterosexual couples; 1 lesbian couple; 2 heterosexual men with SCI; 1 incidentally homosexual female with SCI											
Have a partner	Yes	Yes	Yes	Yes	Yes	Yes	Yes	Yes	Yes	Yes	No
Civil state	Union with civil rite	Union with civil rite	Unmarried	Unmarried	Union with civil rite	Union with civil rite	Cohabitant	Cohabitant	Cohabitant	Union with religious rite	Unmarried
Living with:	Partner	Partner	Alone	Family of origin	Partner	Partner	Partner	Partner	Partner	Partner	Alone
Children	One	One	None	None	None	None	None	One	None	One	None
Education	High school diploma	High school diploma	High school diploma	High school diploma	High school diploma	Bachelor’s degree	Some high school	Bachelor’s degree	High school diploma	Middle school	Some high school
Employment	Retired	Housewife/Husband	Retired	Independent professional	Retired	Unemployed	Independent professional	Employee	Unemployed	Employee	Independent professional
Country of birth	Italy	Cuba	Italy	Italy	Italy	Italy	Italy	Italy	Italy	Italy	Italy
Citizenship	Italy	Italy	Italy	Italy	Italy	Italy	Italy	Italy	Italy	Italy	Italy
Politics	None	None	Right	None	None	Does not know	Right	Left	Center-left	Center	None
Religious affiliation	None	Roman Catholic	None	None	Roman Catholic	Roman Catholic	None	Roman Catholic	None	Roman Catholic	None
6 no religion (54.5%); 5 Roman Catholic (46.5%)											

*The first eight cases are shown paired as couples. Wherever possible, percentages, mean, minimum, and maximum are displayed.*

### Outcome Measures

The results are presented considering all participants and patients and partners groups separately. Table 3 shows the responses to the SIS scale’s items, the SIS scale’s total score and general sexual satisfaction score after injury, the BDI-II, and the BAI before and after the intervention.

**FIGURE 1:**
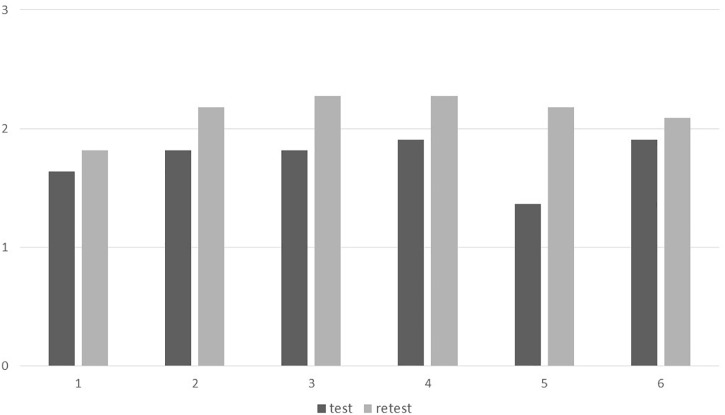
Answers to SIS Scale items before and after intervention. Mean scores to the 6 SIS Scale items before and after intervention (all participants). Vertical axis: 0 = non-existent/very dissatisfying; 1 = decreased/partly dissatisfying; 2 = unchanged/partly satisfying; 3 = increased/very satisfying. Horizontal axis: SIS Scale items – 1 = sexual desire; 2 = importance of sexuality; 3 = sex satisfaction after injury; 4 = sex satisfaction before injury; 5 = enjoy sexuality; 6 = give sexual fulfillment.

All participants (*N* = 11) improved significantly on item 5 (“How are your opportunity and your ability to enjoy sexuality yourself?”) (*z* = −3; *p* < 0.01) (Figure 1), SIS Scale total score (*z* = −2.53; *p* < 0.05), and BAI scores (*z* = −1.99; *p* < 0.05). The effect size was high in all cases (*r* = 0.90, *r* = 0.76, and *r* = 0.60, respectively). No difference was found in the scores for the SIS Scale’s general satisfaction after injury or for BDI.

**Table 3:** Means, standard deviations, *z* value derived from the Wilcoxon test, *p* value, *r* value for all participants and patients and partners groups before and after the intervention program on SIS, BDI-II, and BAI.

	Before Intervention		After Intervention				
	*M*	*SD*	*M*	*SD*	*z*	*p*	*r*
All participants (*N* = 11)							
SIS Scale item 1	1.64	0.81	1.82	0.75	−1.00	0.317	−0.30
(current sexual desire)							
SIS Scale item 2	1.82	0.87	2.18	0.87	−1.63	0.102	−0.49
(current importance of sexuality)							
SIS Scale item 3	1.82	0.75	2.27	0.79	−1.41	0.160	−0.42
(sex satisfaction after injury)							
SIS Scale item 4	1.91	0.70	2.27	0.79	−1.18	0.238	−0.36
(sex satisfaction before injury)							
SIS Scale item 5	1.36	0.67	2.18	0.75	−3.00	0.003^∗∗^	−0.90
(enjoy sexuality)							
SIS Scale item 6	1.91	0.94	2.09	0.70	−1.00	0.317	−0.30
(give sexual fulfillment)							
SIS Scale total score	10.55	2.54	12.82	3.46	−2.53	0.011^∗^	−0.76
SIS Scale general	0.91	0.54	0.00	0.77	0.33	0.739	0.10
satisfaction after injury							
BDI-II	10.82	7.87	8.73	6.78	−0.58	0.562	−0.17
BAI	11.09	7.71	8.00	7.47	−1.99	0.046^∗^	−0.60
(anxiety)							
Patients (*n* = 7)							
SIS Scale item 1	1.43	0.79	1.71	0.76	−1.00	0.317	−0.38
(current sexual desire)							
SIS Scale item 2	1.71	0.95	2.14	1.07	−1.34	0.180	−0.51
(current importance of sexuality)							
SIS Scale item 3	2.14	0.69	2.00	0.82	−0.58	0.564	−218.09
(sex satisfaction after injury)							
SIS Scale item 4	2.14	0.69	2.29	0.76	−0.38	0.705	−0.14
(sex satisfaction before injury)							
(depression)							
SIS Scale item 5	1.29	0.76	2.00	0.82	−2.24	0.025^∗^	−0.85
(enjoy sexuality)							
SIS Scale item 6	2.14	1.07	2.29	0.76	−0.58	0.564	−0.22
(give sexual fulfillment)							
SIS Scale total score	11.00	2.83	12.43	3.87	−1.83	0.068	−0.69
SIS Scale general satisfaction after injury	0.00	0.58	0.28	0.75	−1.00	0.865	0.12
BDI-II	11.43	8.46	9.14	7.31	−0.17	0.157	−0.06
(depression)							
BAI	8.14	6.01	6.43	6.16	1.41	0.317	−0.53
(anxiety)							
Partners (*n* = 4)							
SIS Scale item 1	2.00	0.82	2.00	0.82	0.00	1.000	0.00
(current sexual desire)							
SIS Scale item 2	2.00	0.82	2.25	0.50	−1.00	0.317	−0.50
(current importance of sexuality)							
SIS Scale item 3	1.25	0.50	2.75	0.50	−1.86	0.063	−0.93
(sex satisfaction after injury)							
SIS Scale item 4	1.50	0.58	2.25	0.96	−1.41	0.157	−0.71
(sex satisfaction before injury)							
SIS Scale item 5	1.50	0.58	2.50	0.58	−2.00	0.046^∗^	−1.00
(enjoy sexuality)							
SIS Scale item 6	1.50	0.58	1.75	0.50	−1.00	0.317	−0.50
(give sexual fulfillment)							
SIS Scale total score	9.75	2.06	13.50	3.00	−1.84	0.066	−0.92
SIS Scale general satisfaction after injury	0.25	0.50	0.50	0.58	−0.34	0.197	0.09
BDI-II	9.75	7.80	8.00	6.73	−1.29	0.141	−0.64
(depression)							
BAI	16.25	8.38	10.75	9.71	−1.47	1.000	−0.74
(anxiety)							

*Legend: z = z-value; p = level of significance; r = effect size; SIS Scale’s general satisfaction after injury = difference between post- (SIS Scale item 3) and pre-injury (SIS Scale item 4) values; BDI-II = BDI-II total score; BAI = BAI total score. The effect size was interpreted following Cohen (1992): <0.10, negligible effect; 0.10–0.30, small effect; 0.30–0.50, medium effect; >0.50, large effect).*

A significant effect was found on item 5 (“How are your opportunity and your ability to enjoy sexuality yourself?”) for both patients (*n* = 7; *z* = −2.24; *p* < 0.05) and partners (*n* = 4; *z* = −2; *p* < 0.05) with a high effect size (*r* = 0.84 and *r* = 1, respectively). No effects were found on the total score or general satisfaction after injury of the SIS Scale, BDI-II, or BAI. No significant differences were found between genders or between patients and partners.

## Discussion

The study provided pilot data on the effectiveness of a psychoeducational intervention on the sexual life of a group of in- and outpatients and their partners carried out in a USU-PG. We would like to stress the fact that the first remarkable result obtained by this project (Love & Life) was indeed the realization of an initiative aiming to promote the sexual life of people with SCI within an Italian public health facility. As far as we know, this was the first initiative in Italy aiming to address the issue of improving sexual life and not only to cure sexual dysfunction of people with SCI. Despite the novelty of the proposal, we have had to struggle with deep psychological, cultural, and religious resistance to allow sexuality to be treated not just as a medical dysfunction but as an unavoidable dimension of personal well-being that no trauma can eliminate. Breaking the resistance of people (disabled and not disabled, patients and partners, health personnel, and lay people) to even just imagine that a person with SCI also has the ability to have relationships, experience love, and experience sexual and romantic attraction was a big deal. That said, we have experienced the participation of fourteen people as already a success of our initiative.

### Sex, Education, Religion, and Other Characteristics of the Participants

Although the composition of the personal growth group on sexual life was not determined in any way by a criterion of representativeness of the Italian population with SCI, some characteristics of the sample appear consistent with key facts regarding SCI. For instance, the prevalence of males with SCI in the group reflected the high worldwide male-to-female ratio (4:1, male:female) (Pagliacci et al., 2003; Singh et al., 2014) (The reason why three male patients did not complete the entire socio-demographic questionnaire and/or the outcome measures was because they were not present at the beginning of the first and/or at the end of the last group meetings due to personal circumstances not related to the group activity. We did not administer the questionnaires or outcome measures to them in another context to avoid compromising the homogeneity of the administration setting).

No participant has reached master’s degree level. Only one patient has a bachelor’s degree, and two with some high school. This is consistent with another key fact: SCI is associated with lower rates of school enrollment (World Health Organization [WHO], 2013).

For all the patients, the cause of SCI was traumatic. The mean age when the traumatic event occurred was 38.1, consistently with data provided by Pagliacci et al. (2003) on the Italian SCI population (38.5). Five participants declared that they were Roman Catholic. Two out of them were a lesbian couple with a civil union. The remaining ones declared themselves non-religious. In the sample examined, Catholic affiliation is lower than the national average—74.4% according to Ipsos Public Affairs (2017). Addressing a sexually explicit issue seems to attract more people without religious affiliation or with an unorthodox view (e.g., lesbian couple with civil union), because religions have specific teachings about sex that can condemn masturbation or sexual relationships outside of a heterosexual marriage (Shakespeare et al., 1996; Wendell, 1996; Federici, 2002; Abbott and Howarth, 2005; Graham, 2005; Consortium for Spinal Cord Medicine, 2011; Zilbergeld, 2013).

### Outcome Measures and Effect Size

The effectiveness of the psychoeducational intervention was clearly apparent, showing a high effect size in improving sexual interest and satisfaction and improving the opportunity and ability to enjoy sexuality. A reduction of anxiety was also observed for all participants, although it may not have been related to the psychoeducational intervention. Conversely, the intervention did not appear to significantly reduce levels of depression in either patients or partners. This might be explained by the fact that the level of anxiety observed at the beginning of the first group meeting could be influenced by the context of novelty and sensitivity of the topic being addressed. When the re-test was administered at the end of the last group meeting, the milieu was certainly friendlier and the topic on sexuality less disturbing. Therefore, the reduction of the anxiety levels might be due more to an intervening variable (anxiogenic context) than to effectiveness of the treatment. This could also explain why there was no improvement in the levels of depression that generally tend to positively correlate with anxiety (Beck et al., 1985; Beck and Steer, 1993). The personal growth group on sexual life was, indeed, mainly focused on improving awareness of sexuality, through informative and practical activities, conveyed in part by sexually explicit videos, and through therapy techniques focused on feelings and social relationships. Anxiety and depression might be determined by many other factors (Beck et al., 1985) affecting the quality of life of the patients and, as a consequence, of their partners besides sexual function, interest, and satisfaction. In addition, an efficacious psychotherapeutic treatment for observing reduction in anxiety and depression might require more than 12 meetings over a period of 3 months (Hollon et al., 2006; Shedler, 2010). However, our findings correspond to the study by Harrison et al. (1995) in which anxiety and depression were experienced by the same individuals, and anxiety, but not depression, was related to the sexual dysfunction of woman with SCI.

### Limitations of the Study

Future research might overcome some limitations of the present study. These include, for example, increasing the sample size, given that the sample of participants observed in the present study prevents us from generalizing the results as representative of the Italian population of SCI. This widening of the sample would permit observation about how sex, sexual orientation, education, and other socio-demographic variables affect sexual interest and satisfaction as well as sexual self-esteem. In addition, a randomized controlled trial might reduce bias when evaluating such an intervention. Future studies would be to look at the effect of such an intervention on those in the relative recent post-injury phase as compared to those who have lived with an SCI for many years. It could also be helpful to interrogate the group on how effective they found various components of the intervention to be and what would their suggestions be for an intervention moving forward. The final recommendation is that the most effective way to evaluate such an intervention is a randomized controlled trial.

Since the participants were self-selected and, therefore, probably motivated to make a change in their sexual life, this may have influenced the results after the intervention. Furthermore, as in other observational studies that use self-report questionnaires to collect data, scores could have been easily exaggerated or minimized by the participants completing them. Like all questionnaires, the way the instrument is administered can have an effect on the final score (Bowling, 2005). Another limitation of the study is the social desirability bias (Paulhus, 1991; Bowling, 2005) with self-report measures. In a small group of participants, as in the present pilot data report, answers to the second administration of the outcome measures, after 6 months of meetings on sexuality, might be given to make a good impression and please the group leaders, showing more satisfaction and interest in sexuality and less anxiety. Finally, although the SIS Scale is one of the few sexuality scales that has been used within the SCI population (Abramson et al., 2008), its psychometric properties have been analyzed and provided by only one study (Siösteen et al., 1990) with a relative small sample size and only in its English version.

## Conclusion

Several studies (Tepper, 1999; Fisher et al., 2002; Mona, 2003; Tepper, 2005; Celik et al., 2014; Eglseder and Demchick, 2017) and guides (Tepper, 1997a; Hammond and Burns, 2009; Consortium for Spinal Cord Medicine, 2011) urge that adequate education (Eglseder and Demchick, 2017) and psychological support (Tepper, 1997b) be provided to people with SCI in order to facilitate successful participation in sexual activities. These studies also highlight the need to involve intimate partners in discussions related to sexuality during the rehabilitative process (Eglseder and Demchick, 2017) in an inclusive approach that give women, in the same way as for males, the opportunity to talk with peers with SCI about sexual health, both during initial rehabilitation and after returning home (Kreuter et al., 2008). The first and biggest result of the Love & Life project was just to accomplish in Italy a setting where a psychoeducational intervention met the needs of people with SCI and their partners, providing adequate education and psychological support, involving partners, and creating a space to talk among peers. The effectiveness of intervention also presents positive, clear evidence of the validity of the biopsychosocial model adopted that overcomes a reductive view limiting sexuality to the function/dysfunction of genitalia, phallocentric primacy of sexual pleasure, and sexual attractiveness of only bodily perfection. We do not believe to have solved the complexity of the sexual lives of women (Mona et al., 2009) and men with SCI and their partners, but we hope that the Love & Life project may lead to a new way forward to address this complexity.

## Ethics Statement

This study was approved by the ethics committee of the Department of Philosophy, Social & Human Sciences, and Education, University of Perugia. The project information was disseminated both orally and in paper format. Written informed consent was recorded for each group member on a prepared form by participant signature.

## Author Contributions

SF, FA, PA, and DD conceived and designed the experiments. SF, FA, PA, and DD performed the experiments. MP and SF analyzed the data and contributed materials and analysis tools. SF, FA, MP, PA, and DD contributed to the writing of the manuscript. SF led the development of the original experiments idea. SF, FA, MP, PA, and DD developed the experiments materials, collected the data, and analyzed the initial data. SF and MP interpreted the initial data. SF, FA, MP, PA, DD, IR, and RM supervised the final analysis and the final data interpretation. SF drafted the first manuscript. FA, MP, PA, DD, IR, and RM critically revised the manuscript. SF, FA, MP, PA, DD, IR, and RM approved the final, submitted version.

## Conflict of Interest Statement

The authors declare that the research was conducted in the absence of any commercial or financial relationships that could be construed as a potential conflict of interest.

## Footnotes

1 https://www.unicef.org/media/files/FGMC_2016_brochure_final_UNICEF_SPREAD.pdf

2 https://www.kinseyinstitute.org/research/publications/kinsey-scale.php
