# Supplementary material for: Enhancing Psychological Sexual Health of People With Spinal Cord Injury and Their Partners in an Italian Unipolar Spinal Unit: A Pilot Data Study
Source: Front Psychol. 2019 Apr 5;10:754. doi: 10.3389/fpsyg.2019.00754 (PMC6459940; doi:10.3389/fpsyg.2019.00754)
Supplement: Supplementary file 1 [file Table_1.DOCX]

**Supplementary Material**

to

**Enhancing Psychological Sexual Health of People with Spinal Cord Injury and Their Partners in an Italian** **Unipolar Spinal Unit: A Pilot Data Study**

by

Stefano Federici, Francesco Artegiani, Martina Pigliautile, Paolo Antonelli, Daniele Diotallevi, Innocenza Ritacco, Renée Maschke

In the following tables (S1 and S2), two topic guides for the second and the fifth growth group meetings on sexual life are provided as an example of the psychoeducational activity. As discussed in the “Methods” section of the main text, the activities of the personal growth group on sexual life were structured into two parts: informative and practical. The informative part was addressed every two meetings. The interactive practical part was characterized by group activity—such as “Listing problems” in the phase of “Managing problem” (Table S1) and “Write your sexual fantasies” in the phase “The fantasy box” (Table S2)—which involved all participants simultaneously, as well as individual activity in the group—such as “Sharing problems” and “Defining problem” in the phase of “Managing problem” (Table S1) and “Communicate your sexual fantasy” in the phase “The fantasy box” (Table S2)—which involved one or two participants at a time. The group activity involving informative content was performed every two meetings. The individual activity on a topic of the informative content was carried out for two consecutive meetings to give all participants the opportunity to work on themselves by paying attention to their own body and verbal language, focusing on their own emotions and the here and now of the relationship with the therapist or other group members.

**Table S1. Topic guide of the second meeting of the personal growth group on the sexual life of people with SCI and their partners.**

| **LOVE & LIFE: PERSONAL GROWTH GROUP ON SEXUAL LIFE**  **Topic guide of the second meeting**  **December 30, 2017** | | |
| --- | --- | --- |
| **PHASES AND TIME** | **TOPICS** | **PROCEDURES** |
| **Opening and Introduction**  Time: 20 min | Continuity with the previous meeting | Sharing participants’ thoughts and experiences that emerged after the previous meeting. |
|  | *Me and my new body*: Exploration of erogenous areas and masturbation. | Presentation of the meeting topic and two videos. |
| **Video projection**  Time: 20 min | Rediscovering sexuality after SCI and experiencing non-genital orgasms. | Video projection: *Strange Sex – Surrogate Manhood* (SexAbilityFilms, 2012. <https://youtu.be/9MHO0rwFWqA>).  Time: 11:10 min |
|  | Masturbation and sexual activity with a partner. | Video projection: clip from *Alexander & Sipski* (1993). *Sexuality Reborn: Sexuality Following Spinal Cord Injury*  Time: 2:27 min |
| **Managing problem***  Time: 70 min | Masturbation after SCI | Listing problems: Ask the participants to write three personal issues about masturbation on a piece of paper. For persons with tetraplegia, they will be asked to keep the problems in mind by associating them with a color. |
|  |  | Sharing problems: Invite participants to freely share what they have written. |
|  |  | Defining problem: When a participant has shared the three problems, invite him/her to focus on just one problem. Ask the person to define the problem. For example, ask whether it was present before the SCI, invite to clarify the limits that hinder the resolution (total or partial), investigate the resistance to meeting the sexual needs. |
|  |  | Helpful strategies: Explore together with the group the possible solutions to the problem, based on personal experience, and encourage the participant to identify possible strategies for achieving the objective. |
| *See World Health Organization (WHO). (2016). *Problem Management Plus (PM+)*. Geneva, CH: WHO. |  | Action Plan: Encourage participants to identify a strategy able to bring them closer to the achievement of pleasure or to their own sexual objective.  Ask participants: “*Based on what you have talked about and the reflections you have made, in the period that separates us from the next meeting, what can you do for …*” |
| **Closing**  Time: 10 min |  | Invite all participants to express with a simple word what “I leave here” and what “I take with me” from the meeting just concluded. The word can represent an object, an emotion, an image, etc. |

**Table S2. Topic guide of the fifth meeting of the personal growth group on the sexual life of people with SCI and their partners.**

| **LOVE & LIFE:** **PERSONAL GROWTH GROUP ON SEXUAL LIFE**  **Topic guide of the fifth meeting**  **February 10, 2018** | | |
| --- | --- | --- |
| **PHASES AND TIME** | **TOPICS** | **PROCEDURES** |
| **Opening and Introduction**  Time: 20 min | Continuity with the previous meeting | Sharing participants’ thoughts and experiences that emerged after the previous meeting. |
|  | *Between identity and sexual orientation*: Defining sexuality | Brainstorming: “*What is sexuality for each of you based on the experience in this group?*” |
| **Slide presentation**  Time: 20 min | Sexual fantasies vs. sexual desires | The psychologist presents ten things to know about sexual fantasies, using a slide presentation, picking up on what emerged during the brainstorming. |
| **The fantasy box**  Time: 70 min | Exercise: “*I communicate to you my sexual fantasy”* | Write your sexual fantasies: Each participant writes three sexual fantasies on three different notes that will then be collected in a (fantasy) box.  From another (name) box, in which the names of the participants were previously collected, the name of a person is selected. This person (A) is then invited to select three sexual fantasies from the box and choose one, not sharing this with the others.  Communicate your sexual fantasy:  Having chosen the secret sexual fantasy, A selects a name of one participant, from the box in which the names of the participants were previously collected, who will play the role of partner (B).  A and B are placed facing each other. A describes the scenario in which he/she imagines they (A and B) are both a part of. Then A starts communicating to B the secret sexual fantasy. B can interact with A, asking for explanations, expressing in turn what he/she thinks and feels.  Depending on the opportunities, at the end of A’s work, A and B exchange roles.  If a participant refuses to work, the reason for the refusal will be investigated: what or why he/she is afraid of feeling, participating, etc. |
| **Closing**  Time: 10 min |  | All participants are invited to express with a simple word what “I leave here” and what “I take with me” from the meeting just concluded. The word can represent an object, an emotion, an image, etc. |
